# Psychological Analysis among Goal Orientation, Emotional Intelligence and Academic Burnout in Middle School Students

**DOI:** 10.3390/ijerph17218160

**Published:** 2020-11-04

**Authors:** Pablo Usán Supervía, Carlos Salavera Bordás, Víctor Murillo Lorente

**Affiliations:** 1 Departament of Psychology, Faculty of Human Sciences and Education, University of Zaragoza, Valentín Carderera nº4, 22003 Huesca, Spain; 2 Departament of Psychology, Faculty of Education, University of Zaragoza, Pedro Cerbuna nº12, 50009 Zaragoza, Spain; salavera@unizar.es; 3 Departament of Physiatry and Nursing, Faculty of Health and Sports Sciences, University of Zaragoza, Plaza Universidad nº3, 22002 Huesca, Spain; vmurillo@unizar.es

**Keywords:** adolescents, students, goal orientation, emotional intelligence, burnout

## Abstract

During schooling, students can undergo, for more or less long periods of time, different contextual settings that can negatively affect their personal and academic development, leading them not to meet their academic goals. The main objective of this research responds to examine the relationships between the constructs of goal orientations, emotional intelligence, and burnout in students. **Method:** This research comprised 2896 students from 15 Spanish high schools with ages between 12 and 18 years distributed across male (N = 1614; 55.73%) and female (N = 1282; 44.26%) genders. The measurements were made through Perception of Success Questionnaire (POSQ), the Trait Meta Mood Scale (TMMS-24) and the Maslach Burnout Inventory Student Survey (MBI-SS). **Results:** Results showed links between task orientation, high emotional intelligence levels, and adaptive behaviors and between ego orientation, academic burnout and less adaptive behavior. Similarly, it was shown that emotional intelligence can be used to predict goal-oriented behaviors. **Conclusion:** It is argued that the promotion of task orientation among secondary school students can lead to the adoption of adaptive behaviors and this, in turn, improve the development of students toward academic and personal settings.

## 1. Introduction

During their school stage, students are affected by contextual, situational, and personal circumstances that can have a strong effect on their learning processes, especially at the secondary school level, during adolescence, a vital stage in the life of the individual about to enter adulthood [1].

For more or less prolonged periods of time, some students do not possess, or do not use, the necessary strategies, tools, and skills to meet their academic demands. This triggers feelings and perceptions that undermine their motivation and their wish to continue studying [2], physical and psychic exhaustion [3], negative and unsatisfactory behavior and loss of interest in school [4]. All these circumstances can lead to poor academic performance and even failure and premature school dropouts [5].

### 1.1. Goal Orientation

The achievement goal theory attempts to describe the motives of the people in their life performing different personal behaviors [6]. In this case, this theory would respond to the reasons why students act in one way or another daily at school in achievement environments showing different capacities about their abilities, capabilities and skills which suggests two motivational orientations: one, more self-determined, is task-oriented, while the other, less self-determined, is more ego-oriented.

Those students who are task-oriented have the assumption that the motivation and development of their capacities follow the effort and the dedication that the school demands, and suppose their success at school is related to the assumption of intrinsic motivation in performing tasks [7], coping strategies [8], academic performance and happiness [9], and psychological and emotional well-being [10].

Ego-oriented students aim to show skills, capabilities, and competencies above those shown by their peers, and this is related to extrinsic motivations in undertaking school duties [11], anxiety problems [12], less academic efficacy [13], and even lack of commitment to school activities and dropout [14].

The Achievement Goals Theory considers task or ego orientations depending on how students interpret, respond to and experience goal achievement and all its implications [6].

### 1.2. Emotional Intelligence

The capacity to be able to analyse the emotional information around the environment is called emotional intelligence [15]. Traditionally, the conceptualization of emotional intelligence has been conceptualized in two different ways [16]. The first of them, following the studies of Mayer and Salovey [15], considers emotional intelligence as an inherent cognitive capacity of the person measured by performance-based tests. The second one, the construct of emotional intelligence is considered to be a personality trait and can be measured by questionnaires installed in the lower part of the personality hierarchy levels [17].

Emotionally intelligent people can understand emotions in their immediate environment, understanding their possible causes and effects as well as developing the necessary strategies to regulate or manage different emotional states. In this way, emotional intelligence is constituted by a series of abilities in the person’s subjective wellbeing such as emotional attention, comprehension, and regulation [18]. Therefore, emotional attention responds to the ability to perceive, act and express different emotions adequately; emotional comprehension is the ability to understand emotional changes and moods; and the capacity to regulate emotions adequately is called emotional regulation.

Scientific literature on emotional intelligence in school environments is ample, focusing on different groups and contexts. Most research in recent years analyzed the influence of emotional intelligence in wellbeing of primary school students [19], secondary school students [20] and/or university students [21]. These studies led to significant correlations between emotional intelligence and subjective wellbeing and academic happiness linked with better academic performance [22].

It can be argued that emotions play a fundamental role in the adaptation of adolescents in their school environment, helping them to manage all situations with the potential to affect their personal wellbeing, academic motivation, and academic performance, among others [18].

### 1.3. Academic Burnout

By adopting more non-adaptative behaviors, some students can lose interest in, and no longer feel committed to, their studies, while doubts and/or contradictions about their own personal capabilities arise, which prevent them from moving forward. As noted, this can lead some students to a complete lack of motivation, and even to drop out of school [23]. The presence of these personal symptoms is well known as *academic burnout* [24,25] and the three main dimensions are emotional exhaustion, cynicism, and self-efficacy. On the other hand, emotional exhaustion is associated with the physical and emotional weariness that students might feel, more or less persistently, during their academic lives; cynicism refers to relaxed attitude, indifference and a little interest in their studies and finally, self-efficacy relates to the student’s attitude toward the academic tasks, demands and duties. Finally, academic burnout is associated with high levels of stress [26], low levels of vigor and involvement with and dedication to academic duties [27], low levels of self-efficacy [28] and low levels of academic performance [11].

### 1.4. Goal Orientation, Emotional Intelligence and Academic Burnout

Scientific literature has paid little specific attention to the relationship between these two constructs in students, but some studies exist that approach the issue from a different perspective.

Currently, goal orientation and emotional intelligence are used interchangeably. Emotional intelligence is regarded as a way to interact with our environment, and the motivations of the subject play a very important role in this; as such, emotions are an integral part of motivation, insofar as this triggers goal-oriented behaviours [29]. As pointed out by Fernández, Anaya and Suárez [30], motivational systems and emotional intelligence interact and support one another in pursuance of the desired goal, and to the detriment of interdependent positions. Buck, Powers and Hull [31] found positive relationships between task orientations and the three dimensions that constitute emotional intelligence, as well as with greater commitment to the school task and academic enjoyment. Froiland and Worrell [32], in analyzing a sample of primary and secondary school students, found a relationship between these variables and the student’s engagement with the school. Gargallo, Campos and Almerich [33] highlighted the fact that more intrinsically and emotionally intelligent students performed better and showed greater levels of self-efficacy.

By combining the two goal-oriented variables and academic burnout, several studies have related achievement goals and levels of burnout by showing that motivational task-oriented achievement and academic self-efficacy converge in more self-determined behavior patterns [34] while ego-oriented achievement goals are related to emotional exhaustion and cynicism in a lower self-determined behavior patterns [27].

Finally, all studies agree that emotional intelligence and academic burnout are negatively correlated [35,36]. As an example, in a recent study with the same kind of population as in our research, it was determined that students with high emotional intelligence were less likely to experience school anxiety and more likely exhibiting resilience which in turn, reduced the risk of school burnout [37]. In any case, the greater the degree of emotional intelligence, the lower the levels of emotional exhaustion and cynicism. Conversely, the greater the level of emotional intelligence, the higher the level of academic self-efficacy and subjective wellbeing [36].

### 1.5. Study Objective

Based on the previous arguments, and following Cera, Almagro, Conde and Sáenz-López [38] it can be argued that few studies have specifically analyzed our target variables in the primary and secondary school environment. Therefore, to increase the scientific literature is needed more studies to approach the understanding of the relationship between our target variables, with the ultimate aim of improving the students’ academic and personal development in school environments. The main objective of this research responds to analyze and examine the relationships between the variables of goal orientations, emotional intelligence, and burnout in students.

Four hypotheses are presented:

**Hypotheses** **1.** *Task-oriented students present greater levels of emotional intelligence and lower of academic burnout, following more adaptive behaviour.*

**Hypotheses** **2.** *Ego-oriented students present greater levels of academic burnout and lower of emotional intelligence, following less adaptive behaviours.*

**Hypotheses** **3.** *Emotional attention, comprehension and regulation can be used to predict task-oriented behaviors.*

**Hypotheses** **4.** *Physical/emotional exhaustion related to academic burnout can predict ego-oriented behaviors.*

## 2. Materials and Methods

Participants: This study was composed of 2896 students, both male (N = 1614; 55.73%) and female (N = 1282; 44.26%) with ages ranging from 12 to 18 years (M = 14.78; DT = 1.71) belonging to 15 public schools which were selected by simple random sampling. The students belonging to the different classes of the schools responded to the inclusion criterion in filling the questionnaires as soon as they knew how to read and understand the items of the questionnaires. Incomplete questionnaires (84) were discarded to be include in the sample, including those from students who decided to drop out half way and students with cognitive disorders who could not fully understand the questionnaire were excluded from the study. The missing data was around 3%, taking valid and suitable questionnaires around 97%.

Measurements: The following questionnaires were completed by the research participants: Perception of Success Questionnaire (POSQ) [39] translated into Spanish language by Martínez, Alonso and Moreno [40]. Composed of 12 items and two subvariables: task-oriented (e.g., “When I’m in class, I perform to the best of my ability”); and ego-oriented (e.g., “When I’m in class, I feel successful when I show the teacher and my classmates that I am the best”) distributed on a five-point Likert scale in which the students showed their degree of adequacy with the items presented where “1. Strongly disagree” was the minimum mark and “5. Strongly agree” the maximum mark. Cronbach’s alpha values was 0.86 for task-oriented and 0.83 for ego-oriented, respectively

Trait Meta Mood Scale-24 (TMMS-24) [41] adapted in a shortened version by Fernández-Berrocal, Extremera and Ramos [42]. The scale is composed of three subvariables: emotional attention (α = 0.79) (e.g.,: ‘I pay much attention to my feelings’); emotional comprehension (α = 0.83) (e.g., ‘I know how to label my emotions´); and emotional regulation (α = 0.82) (e.g., ‘Even if I am not feeling well, I try to have positive thoughts’) distributed on a five-point Likert scale in which the students showed their degree of adequacy with the items presented where “1. Strongly disagree” was the minimum mark and “5. Strongly agree” the maximum mark. 

Maslach Burnout Inventory—Student Survey (MBI-SS) [24]. The inventory in Spanish language consists of 15 items and three subvariables: physical/emotional exhaustion (α = 0.82) (e.g., “Studying or going to class all day is exhausting”); cynicism (α = 0.80) (e.g., “I have become less enthusiastic about my studies”) and self-efficacy (α = 0.79) (e.g., “I feel stimulated when I achieve my study goals”). The inventory was distributed on a five-point Likert scale where “1. Strongly disagree” was the minimum mark and “5. Strongly agree” the maximum mark.

Procedure: Prior to conducting this study, approval was obtained from the participating ESO schools and from the students’ parents/guardians. In agreement with the different educational schools, the questionnaires were handed out to all groups. Parents/guardians voluntarily participated through Helsinki Declaration [43] ethical guidelines. The research protocol was endorsed by Psychology and Sociology Department, Universidad de Zaragoza. The research code is (4620). The schools were in charge of telling us the best day to pass the questionnaires to their students. On that day, we passed the questionnaires to the same school in the different classes (1–4 grade) with the help of their tutors in a single class. All the questionnaires were totally anonymous and voluntary and they could even abandon it at any time if they wished not to continue with it.

Data analysis: To carry out the statistical analysis in the research, the description of the variables and sociodemographic data of the participants was carried out in matters such as sex, age and academic year. In turn, a correlational analysis of the three main constructs of the study was carried out. On the other hand, a stepwise multiple regression (showing the last step) was used with the intention of predicting goal orientations (task and ego) on the variables of emotional intelligence and burnout. For these procedures, IBM SPSS v26.0 (IBM, Armonk, NY, USA) was used. Finally, an equation model was performed between the three variables described under the maximum likelihood method with AMOS v24 (IBM, Armonk, NY, USA). For all these procedures adopted a *p* ≤ 0.05 significance level and a 95% confidence.

## 3. Results

The statistical results of the research are described below.

### 3.1. Demographic Variables

The research comprised 2896 students, distributed in male (N = 1614; 55.73%) and female (N = 1282; 44.26%) adolescents, who were ranging from 12 to 18 years (M = 14.78; DT = 1.71), from 15 public secondary schools (Table 1).

### 3.2. Descriptive Variables

In Table 2 we can see how different results were found by gender. Approaching to gender, concerning goal task-oriented, emotional attention and self-efficacy are more pronounced in females. Concerning goal ego-oriented, emotional comprehension and regulation and cynicism values, these variables are in males.

### 3.3. Correlational Analysis of Goal Orientation, Emotional Intelligence and Burnout

Then, a correlational analysis was carried out with the corresponding significant relationships of the variables (see Table 3).

Task orientation is positively correlated with all three dimensions of emotional intelligence—attention (*r* = 0.383), comprehension (*r* = 0.378) and regulation (*r* = 0.355)—and in a negative way with burnout variables as exhaustion (*r* = −0.182) and cynicism (*r* = −0.434). Besides, ego orientation goal was positively correlated, albeit less strongly, with the three dimensions that constitute emotional intelligence and academic burnout.

Finally, emotional comprehension and regulation correlated negatively with physical/emotional exhaustion and cynicism. However, academic self-efficacy correlated positively with emotional comprehension (*r* = 0.331) and regulation (*r* = 0.388).

### 3.4. Regression Analysis of Emotional Intelligence and Academic Burnout on Goal Orientation

In this procedure, the variables of emotional intelligence and academic burnout will be used as possible predictors of goal orientation (task and ego). The results of the final step of the multiple regression analysis are shown in Table 4 and Table 5, introducing the variables that have a significant effect on the task-oriented and ego-oriented goals.

In this procedure created for goal orientation, the predictor variables used were emotional attention, comprehension and regulation, academic self-efficacy (emotional intelligence) and cynicism (with a negative value) (academic burnout). Nagelkerke’s *R*^2^ obtained *a* value 0.342 for task-oriented goal (Table 4).

Concerning ego orientation (Table 5), the predictor variables used were emotional comprehension, academic self-efficacy and physical/emotional, and the adjustment value was *R^2^* = 0.077.

### 3.5. Goal Orientation, Emotional Intelligence and Academic Burnout Structural Equation Model

**Figure 1:**
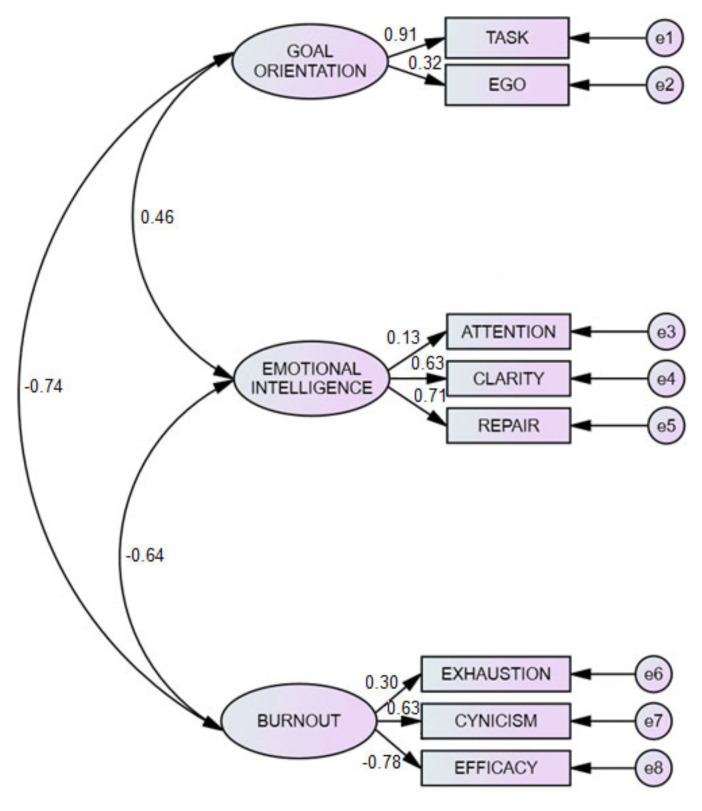
Goal orientation, emotional intelligence and academic burnout structural equation model.

Finally, Figure 1 shows the result of the analysis undertaken with structural equations and the Maximum Likelihood Method, which confirms the suitability of the model and the constructs considered herein. The model indicates a negative correlation of goal orientation and burnout (*r* = −0.64). Furthermore, goal orientations are correlated in a positive relation with emotional intelligence (*r* = 0.46) and academic burnout negatively correlated with emotional intelligence (*r* = −0.74). The adequacy and validity of the structural equations model carried out was endorsed by the appropriate indices: x^2^ (17) = 52.396, *p* < 0.001; x^2^/gl = 3.082; CFI = 0.96; NFI = 0.95; TLI = 0.90; RMSEA = 0.070, IC = 95% (0.052–0.089).

## 4. Discussion

The purpose of this research was to examine the relation among goal orientation (task and ego), emotional intelligence and academic burnout in adolescent students. Four hypotheses were put forward: 

First, that task-oriented students present greater levels of emotional intelligence and lower of academic burnout, following adaptive behaviors.

The supposition was completely confirmed. Our statistical results showed the variables are strongly correlated. In this way, the task orientation of the adolescent students who have the belief that success comes from dedication and effort toward school demand is positively related to emotional attention, comprehension, and regulation, and negatively correlated with the less self-determined physical/emotional exhaustion and cynicism, subvariables pertaining to burnout construct.

The scientific literature shows studies that maintain the line of the results found between the relationship between goal orientation and emotional intelligence. Buck, Powers and Hull [31] advocate positive relationships with the three dimensions that constitute emotional intelligence, along with greater motivation and academic enjoyment. Ferriz, Sicilia and Sáenz [44] highlight the relation between high motivation and emotional intelligence, leading to better academic performance. Froiland and Worrell [32], in analyzing a group of primary and secondary school students, found a relationship between emotional intelligence and task orientation, which are also in relation to the student’s commitment to the school. In the same vein, Cera, Almagro, Conde and Sáenz-López [38] add that greater emotional control leads to less self-perceived stress in adolescents. Therefore, it is argued that task-oriented adaptive behaviors and good levels of emotional intelligence lead to greater psychological and emotional wellbeing and less academic burnout in adolescent students [10].

The second hypothesis stated that ego-oriented students present greater levels of academic burnout and lower levels of emotional intelligence, following less adaptive behaviors.

We were only able to partially demonstrate this hypothesis. On the one hand, there was no negative correlation that we predicted in the relationship between emotional intelligence and ego orientations, even showing that the variables were related in a positive way. On the other, the results found maintain the line that the orientation toward the ego was related to academic burnout and non-adaptive behaviors of adolescent students.

Some studies reach similar conclusions concerning the relationship between ego orientation, academic burnout and non-adaptive behaviors. Caballero, Bresó and González [23] argue that less motivated and ego-oriented students suffer greater physical/emotional exhaustion and cynicism. DeFreese and Smith [11] claim that the relationship between goal orientation and extrinsic motivations leads to less academic commitment and engagement. Estrada et al. [35] take into consideration school commitment and task-confronting strategies that lead to non-adaptive behaviors and less psychological wellbeing.

Therefore, the results of the research showed that ego orientation does not have to be related with low levels of emotional intelligence. Following to Saies, Arribas, Cecchini, De Cos and Otaegi [45] extrinsic orientations do not necessarily involve negative attitudes. In this regard, the metacognitive ability of subjects to choose themselves the positive factors of two goal orientations (task and ego) is important when carrying out their school tasks. According to Pekrun [46] managing positive emotions such as hope of success, anticipated enjoyment, subjective normative ability, pride, the feeling of having one’s value recognized by significant third parties, etc. can be regarded as positive extrinsic motivations which complement and, to some extent replace, intrinsic motivations. Therefore, students can be motivated complementary towards task orientation and ego orientation according to their motives, aims and reasons for carrying out any activity in any academic and personal situation [45,46].

In this vein, various studies point out that task orientation has a stronger correlation with emotional intelligence than ego, but that the latter correlation exists [47,48]. A relationship between non-adaptive conducts and ego orientation and academic burnout is attested, but the expected negative correlation between ego orientation and low levels of emotional intelligence has not. It is thus shown that in order to pursue their ends and meet their academic targets, students can use their emotional intelligence to find a balance between positive and negative elements in both goal orientations [49].

The third hypothesis stated that emotional attention, comprehension, and regulation can be used to predict task-oriented behaviors. This hypothesis was fully demonstrated, and the exercise also showed that the most determinant factor in this regard was self-efficacy.

Few studies analyzed the predictor variables of goal orientation, but approach the issue from a different perspective. Pérez’s [50] analysis of the impact of emotional intelligence on goal orientation argues that emotional comprehension and regulation can be used to predict goal orientation, in line with our own results, which also include emotional attention. Cera et al. [38] found that the dimensions of emotional intelligence can predict more self-determined goal orientations. Inam, Nomaan and Abiodullah [51] suggest that intrinsic motivation, emotional intelligence, and life satisfaction predict self-determined goal orientations.

Finally, the fourth hypothesis stated that physical/emotional exhaustion related to academic burnout can be used to predict ego-oriented behaviors. This hypothesis was not corroborated, although it was shown that physical/emotional exhaustion, emotional comprehension and self-efficacy can be used to predict ego-oriented behavior, in line with previous conclusions, i.e., ego-oriented behaviours carry with them negative implications, although subjects can use them to meet their ends and their academic targets. Schaufeli and Salanova [25] advocate that exhaustion and cynicism can be used to predict extrinsic motivations. In the same vein, Gutiérrez, Ruiz and López [52] emphasize the impact of academic burnout on academic motivation.

## 5. Conclusions

Based on these results, the effect of goal orientation, emotional intelligence and academic burnout in education seem to be beyond discussion. In addition to other personal and contextual variables, these features constitute a psychological profile that could have an important impact on the academic performance and commitment to school. For example, these findings may lead to the recognition in educators of exhaustion situations and maladaptive responses in their students that affect their school learning as well as poor school performance or lack of motivation in tasks to promote adaptive behaviors such as goal orientation toward the task or emotional intelligence that help prevent burnout or cynicism. At the same time, educational programs carried out by educational professionals (teachers, psychologists, educational communities, etc.) can help improve the variables studied in our study for optimal personal and academic development. Therefore, to advocate for adequate personal and academic development of adolescent students, it is essential to address these issues that to a large extent, will also affect their school development [53].

Finally, as study limitations, the cross-sectional design can be highlighted by taking the data in the schools at a unique moment. This can vary in different trimesters and can affect the scores on the variables studied depending on the academic performance, personal and/or social situation with their classmates or even their happiness or boredom in class. Likewise, the schools responded to a random selection not being able to take all areas of the city with its own features despite the large number of subjects involved in the research. Besides, the variations in scores in the variables of goal orientations, emotional intelligence and academic burnout may change from year to year and it is appropriate to opt for longitudinal studies that measure the evolution of the constructs in subsequent investigations.

## Figures and Tables

**Table 1:** Socio-demographic data by variables of gender, age, and school year.

Socio-Demographic Variables		N	%
Gender (male–female)	Male	1614	55.73
	Female	1282	44.26
Age (12–18)	12	447	15.43
	13	543	18.75
	14	553	19.09
	15	677	23.37
	16	476	16.43
	17	154	5.31
	18	46	1.58
School year (1–4 grade)	Year 1	698	24.10
	Year 2	745	25.72
	Year 3	867	29.93
	Year 4	586	20.23

**Table 2:** Goal orientation, emotional intelligence, and academic burnout descriptive variables.

Psychological Variables	Total		Male		Female	
	*x*	*sd*	*x*	*sd*	*x*	*sd*
Goal Task-oriented	3.80	0.80	3.71	0.82	3.91	0.76
Goal Ego-oriented	2.89	1.03	2.95	0.98	2.82	1.08
Emotional attention	3.44	0.75	3.35	0.76	3.55	0.72
Emotional comprehension	3.41	0.72	3.50	0.70	3.31	0.74
Emotional regulation	3.56	0.75	3.62	0.70	3.49	0.79
Physical/emotional exhaustion	3.19	0.96	3.17	1.01	3.22	0.91
Cynicism	2.14	1.06	2.29	1.08	1.97	0.99
Self-efficacy	3.52	0.77	3.49	0.80	3.55	0.72

X: Mean/SD: Standard Deviation.

**Table 3:** Goal orientation, emotional intelligence, and academic burnout correlation analysis.

Psychological Variables	1	2	3	4	5	6	7	8
(1) Goal Task-oriented	1							
(2) Goal Ego-oriented	0.289 **	1						
(3) Emotional attention	0.383 **	0.100 **	1					
(4) Emotional comprehension	0.378 **	0.029 **	0.155 **	1				
(5) Emotional regulation	0.355 **	0.093 **	0.255 **	0.0451 **	1			
(6) Physical/emotional exhaustion	−0.182 **	0.132 **	0.100 **	−0.136 **	−0.198 **	1		
(7) Cynicism	−0.434 **	0.164 **	0.047	−0.172 **	−0.251 **	0.328 **	1	
(8) Self-efficacy	0.520 **	0.256 **	0.060	0.331 **	0.388 **	−0.158 **	−0.489 **	1
*X*	3.80	2.89	3.44	3.41	3.56	3.19	2.14	3.52
*SD*	0.80	1.03	0.75	0.72	0.75	0.96	1.06	0.77
*Cronbach’s* α	0.86	0.83	0.79	0.83	0.82	0.82	0.80	0.79

** Significant correlation at 0.01 (bilateral); X: Mean/SD: Standard Deviation.

**Table 4:** Emotional intelligence and academic burnout on goal orientation (task).

Psychological Variables	*B*	s.e.	*R* ^2^	*t*	Sig.
Constant	2.172	0.296	0.342	7.334	0.000
Attention	0.095	0.035		1.672	0.006
Comprehension	0.096	0.035		2.701	0.007
Regulation	0.093	0.030		3.608	0.002
Self-efficacy	0.363	0.035		10.303	0.000
Cynicism	−0.165	0.024		−6.780	0.000

Excluded variables: Physical/emotional exhaustion (academic burnout). s.e.: standard error; sig.: significative or not.

**Table 5:** Emotional intelligence and academic burnout on goal orientation (ego).

Psychological Variables	*B*	s.e.	*R* ^2^	*t*	Sig.
Constant	1.082	0.243	0.077	4.448	0.000
Comprehension	0.117	0.049		2.377	0.018
Self-efficacy	0.323	0.047		6.953	0.000
Exhaustion	0.086	0.035		2.448	0.015

Excluded variables: Emotional attention and regulation (emotional intelligence)/Cynicism and self-efficacy (academic burnout).
